# Supervised segmentation framework for evaluation of diffusion tensor imaging indices in skeletal muscle

**DOI:** 10.1002/nbm.4406

**Published:** 2020-10-01

**Authors:** Laura Secondulfo, Augustin C. Ogier, Jithsa R. Monte, Vincent L. Aengevaeren, David Bendahan, Aart J. Nederveen, Gustav J. Strijkers, Melissa T. Hooijmans

**Affiliations:** ^1^ Department of Biomedical Engineering and Physics, Amsterdam University Medical Centers University of Amsterdam The Netherlands; ^2^ Aix Marseille Universite, Universite de Toulon, CNRS, LIS Marseille France; ^3^ Aix Marseille Universite, CNRS, CRMBM Marseille France; ^4^ Department of Radiology and Nuclear Medicine, Amsterdam University Medical Centers University of Amsterdam The Netherlands; ^5^ Radboud Institute for Health Sciences, Department of Physiology Radboud University Medical Center Nijmegen The Netherlands

**Keywords:** applications, diffusion tensor imaging (DTI), methods and engineering, muscle, musculoskeletal, post‐acquisition processing, quantitation

## Abstract

Diffusion tensor imaging (DTI) is becoming a relevant diagnostic tool to understand muscle disease and map muscle recovery processes following physical activity or after injury. Segmenting all the individual leg muscles, necessary for quantification, is still a time‐consuming manual process. The purpose of this study was to evaluate the impact of a supervised semi‐automatic segmentation pipeline on the quantification of DTI indices in individual upper leg muscles. Longitudinally acquired MRI datasets (baseline, post‐marathon and follow‐up) of the upper legs of 11 subjects were used in this study. MR datasets consisted of a DTI and Dixon acquisition. Semi‐automatic segmentations for the upper leg muscles were performed using a transversal propagation approach developed by Ogier et al on the out‐of‐phase Dixon images at baseline. These segmentations were longitudinally propagated for the post‐marathon and follow‐up time points. Manual segmentations were performed on the water image of the Dixon for each of the time points. Dice similarity coefficients (DSCs) were calculated to compare the manual and semi‐automatic segmentations. Bland‐Altman and regression analyses were performed, to evaluate the impact of the two segmentation methods on mean diffusivity (MD), fractional anisotropy (FA) and the third eigenvalue (*λ*_3_). The average DSC for all analyzed muscles over all time points was 0.92 ± 0.01, ranging between 0.48 and 0.99. Bland‐Altman analysis showed that the 95% limits of agreement for MD, FA and *λ*_3_ ranged between 0.5% and 3.0% for the transversal propagation and between 0.7% and 3.0% for the longitudinal propagations. Similarly, regression analysis showed good correlation for MD, FA and *λ*_3_ (*r* = 0.99, *p* < 60; 0.0001). In conclusion, the supervised semi‐automatic segmentation framework successfully quantified DTI indices in the upper‐leg muscles compared with manual segmentation while only requiring manual input of 30% of the slices, resulting in a threefold reduction in segmentation time.

## Graphical abstract

The performance of a semi‐automatic segmentation framework was compared to manual segmentation for quantification of DTI indices in individual upper leg muscles in a longitudinal study set‐up. The main advantage is a 3‐fold reduction in segmentation time at baseline and 10‐fold reduction for 3 time points. The average DSC value for all analyzed muscles over all time points was 0.92 ± 0.01. Regression analysis and Bland‐Altman analysis showed that the agreement and correlation was good for MD,FA and higher for *λ*_3_.

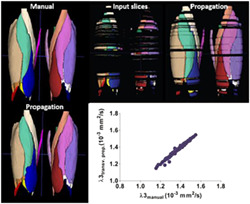

**Abbreviations**
BFL: biceps femoris long head muscle
BFS: biceps femoris short head muscle
CNN: convolutional neural network
DSC: Dice similarity coefficient
DTI: diffusion tensor imaging
FA: fractional anisotropy
G: gracilis muscle
IVIM: intra‐voxel incoherent motion
LoA: limits of agreement
MD: mean diffusivity
RF: rectus femoris muscle
S: sartorius muscle
SM: semimembranosus muscle
SNR: signal‐to‐noise ratio
SPAIR: spectral attenuated inversion recovery
SPIR: spectral pre‐saturation with inversion recovery
SSGR: slice selective gradient reversal
ST: semitendinosus muscle
VI: vastus intermedius muscle
VL: vastus lateralis muscle
VM: vastus medialis muscle
*λ*_3_: eigenvalue 3

## 1 INTRODUCTION

MRI is extensively used in clinical evaluations of skeletal muscle to assess the location, extent and severity of several pathological conditions.^1^, ^2^ The common contrast mechanisms in clinical skeletal muscle scans consist of *T*_1_‐weighted contrast to assess muscle anatomy and fat infiltration, and sequences that employ *T*_2_‐weighted contrast to characterize muscle edema. Over the years, advances in MRI techniques and post‐processing have expanded the MRI toolbox for assessing healthy and diseased muscle composition, architecture, perfusion and function in a quantitative manner, leading to what is known as quantitative MRI.

A quantitative MRI technique that has received considerable attention is diffusion tensor imaging (DTI). DTI probes the self‐diffusivity of water in tissue and its orientational dependence allows for three‐dimensional reconstructions of muscle fiber architecture.^3^, ^4^ Quantitative DTI‐derived indices are heavily dependent on tissue integrity and have been shown to change in a variety of skeletal muscle pathologies and injuries.^5^, ^6^ Furthermore, in sport medicine, DTI has shown promise as a relevant tool to understand the muscle recovery process that follows physical activity and after injury.^7^, ^8^ Our group investigated changes in DTI indices in the upper leg muscles during recovery after a marathon.^9^, ^10^ Distinct changes in DTI indices were detected in muscles that appeared normal on *T*_2_‐weighted images, demonstrating the added value of DTI as a sensitive readout of muscle integrity.

The analysis of DTI acquisitions requires many post‐processing steps for calculation of the DTI indices, which have been almost entirely automated—for more details see, eg, Reference ^11^. However, segmentation of all the leg muscles, necessary for quantification, is a time‐consuming manual process, in which the accuracy and reproducibility may be operator dependent.^12^ Therefore, there is great need for (semi‐) automatic segmentation tools in the context of quantitative imaging of skeletal muscle. Recently, some (semi‐) automatic approaches have been proposed for skeletal muscle^12^, ^13^, ^14^, ^15^, ^16^, ^17^, ^18^, ^19^, ^20^ and showed good correspondence, reflected by high Dice similarity coefficient (DSC) values, with manual segmentation. However, these approaches only focused on either a partial volume of the muscles or entire muscle groups rather than the full volume of individual muscles. Moreover, so far little is known about the impact that (semi‐) automatic segmentation approaches may have on quantification of imaging outcome measures in skeletal muscle, especially on DTI analysis.

The purpose of this paper was to evaluate a supervised muscle segmentation framework, previously developed by Ogier et al,^21^, ^22^ for the quantification of DTI indices in individual muscles. Using this tool, we aimed to assess the accuracy, feasibility and impact on the quantification of DTI indices considering manual segmentation as the ground truth. Also, we aimed to assess the corresponding reduction in work load compared with manual segmentation.

## 2 METHODS

### 2.1 Participants and study set‐up

The study set‐up and the MRI protocol of the study are reported in detail by Hooijmans et al.^10^ For this study we included 11 subjects who competed in the marathon in Amsterdam (The Netherlands) on 15 October 2017. The participants were 51 ± 6‐year‐old males of average fitness level. MRI examinations were obtained at three time points: (1) baseline (1 week prior to the marathon), (2) post‐marathon (24‐48 h after the marathon) and (3) follow‐up (2 weeks after the marathon). The study was approved by the local medical ethics committee and all participants signed written informed consent.

### 2.2 MRI protocol

Participants received an MRI examination of both upper legs on a 3 T MR scanner (Ingenia, Philips, Best, The Netherlands) using a 16‐element anterior body receive coil and a 12‐channel table top coil. Subjects were positioned in a feet‐first supine position in the scanner. The data were acquired in three transversal stacks with 30 mm overlap, covering 498 mm proximal to distal with a field of view (FOV) of 480 × 276 mm^2^. The total duration of the scan protocol was 48 min and included diffusion‐weighted spin‐echo Echo‐planar imaging for DTI parameter estimations (SE‐EPI, bandwidth = 31 Hz/pixel, *T*_R_ = 5,000 ms, *T*_E_ = 57 ms, matrix = 160 × 92, voxel size = 3 × 3 × 6 mm^3^, number of slices = 31, SENSE = 1.9, *b*‐values (s/mm^2^)/no. of directions = 0/1, 1/6, 10/3, 25/3, 100/3, 200/6, 400/8 and 600/12, fat suppression: combination of a spectral pre‐saturation inversion recovery (SPIR) pulse, spectral attenuated inversion recovery (SPAIR) pulse and slice selective gradient reversal (SSGR)). A combination of the three suppression techniques was used to suppress the signal from both the olefinic and aliphatic fat peaks. The signal from the olefinic fat peak at 5.3 ppm was suppressed with a non‐selective SPIR pulse, whereas the aliphatic main peak at 1.3 ppm was suppressed using a combination of SPAIR and SSGR.^23^, ^24^, ^25^ In the SSGR consecutive opposite slice selective gradients are applied on consecutive radiofrequency pulses, which are centered on the water section, resulting in a reduction of the excited fat section and consequently of the fat signal intensity.^26^ For anatomical reference a four‐point mDIXON fast field echo sequence was used (MS‐FFE, bandwidth = 434 Hz/pixel, *T*_R_ = 210 ms, *T*_E_ = 2.60, 3.36, 4.12 and 4.88 ms, matrix = 320 × 184, voxel size = 1.5 × 1.5 × 6 mm^3^, number of slices = 31, SENSE = 2), a noise map to calculate signal‐to‐noise ratios (SNRs) (SE‐EPI without diffusion weighting, *T*_R_ = 5000 ms, *T*_E_ = 57 ms, matrix = 160 × 160, voxel size = 3 × 3 × 6 mm^3^, number of slices = 31, SENSE = 1.9, SPIR/SPAIR/SSGR), as well as a multi‐turbo spin‐echo sequence for quantitative water *T*_2_ mapping and a fat‐suppressed *T*_2_‐weighted scan to assess acute muscle damage. For the purpose of this study, the diffusion‐weighted spin‐echo EPI, noise and Dixon acquisitions were considered.

### 2.3 DTI parameter estimations

MR images were analyzed using QMRITools for Wolfram Mathematica (https://mfroeling.github.io/QMRITools). Diffusion data were de‐noised using a principal component analysis (PCA) noise algorithm, and corrected for motion and eddy currents using affine registration (elastix: http://elastix.isi.uu.nl). Second, the diffusion data were registered to the anatomical space using sequential rigid and B‐spline registration to correct for EPI distortions. The diffusion tensor was calculated using an intra‐voxel incoherent motion (IVIM)‐based iterative weighted linear least squares algorithm (iWLLS).^27^ By using IVIM correction, an anisotropic pseudo‐diffusion component was modeled in addition to the standard diffusion tensor, to remove the perfusion biases in the diffusivity estimation,^28^ as explained in the study by Hooijmans et al.^10^ The third eigenvalue (*λ*_3_), mean diffusivity (MD) and fractional anisotropy (FA) were used as outcome parameters and shown as mean values over the full muscle volume. Previous studies^10^ have shown that *λ*_3_ is the most sensitive DTI parameter to training, whereas MD and FA show the highest and the lowest repeatability respectively.^29^ SNR was defined as the mean of the signal in a muscle region of interest divided by the standard deviation of the noise (*σ*). Muscles with an SNR value below 15 were excluded from the Bland‐Altman and linear regression analyses.

### 2.4 Manual segmentation

Manual segmentation was considered the ground truth for the comparisons with the results of the supervised automatic segmentations. Manual segmentations of 10 muscles in both upper legs, ie biceps femoris short head (BFS), biceps femoris long head (BFL), semitendinosus (ST), semimembranosus (SM), gracilis (G), sartorius (S), vastus medialis (VM), vastus lateralis (VL), vastus intermedius (VI) and rectus femoris (RF) muscles were performed by two experts in muscle anatomy (MH, JM) on the out‐of‐phase Dixon images in ITK‐SNAP (www.itksnap.org). These segmentations were done for all subjects at baseline and the two post‐marathon time points. Delineation of the muscles was done avoiding fascia and subcutaneous fat tissue. Additionally, they were eroded by one pixel to avoid partial volume effects due to fat tissue. The segmentations were subsequently registered (elastix: http://elastix.isi.uu.nl) to the DTI acquisitions to correct for misalignments between the Dixon and the lower‐resolution diffusion scans. The time required for the manual segmentation of one dataset including the whole set of muscles in two legs for a single time point was on average 420 min.

### 2.5 Supervised segmentation

**FIGURE 1:**
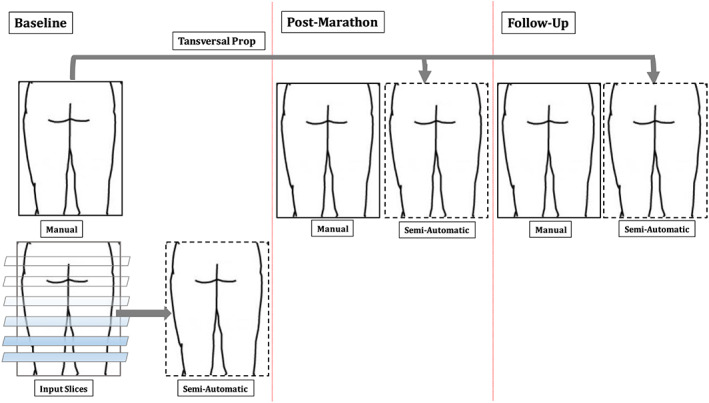
Schematic of the study set‐up. DTI measurements were performed at 3 time points, i.e. at baseline, 24‐48 hrs. post‐marathon and at a 2 weeks follow‐up. Manual segmentations were performed for 10 muscles in both upper legs at all 3 time points. At baseline, a supervised semi‐automatic segmentation was performed based on a selected set of manually segmented slices (transversal propagation). For the post‐marathon and the follo‐up time points the segmentation was automatically propagated without further manual input (longitudinal propagation)

Segmentation of the muscles was performed on the out‐of‐phase Dixon images, as schematically illustrated in Figure 1. The first step of the supervised segmentation started with a manual segmentation of the muscles in a limited number of slices within the full muscle volume, whereas in the study by Ogier et al^21^ muscles were delineated in the muscle belly in two slices 10 cm above the knee and 10 cm below the hip, respectively. Additional manual segmentations were performed in slices for which the muscle changes drastically in size by more than 30 pixels, and at the muscle's origin and insertion. Subsequently, an N4 bias correction algorithm^30^ was used on all data sets. After this, the segmentation was automatically completed based on a propagation algorithm, named transversal propagation, which uses both shape information from the initial manual segmentations and grayscale anatomical information to follow the anatomical variations of muscles along the leg, as described by Ogier et al.^21^ Because of the differences in size and shape between the 10 upper‐leg muscles and between subjects, the number of manually segmented slices differed between muscles and subjects (see Section 3). We refer to the segmentation of the muscles in a single subject as the transversal propagation step. For the post‐marathon and follow‐up time points, the manual segmentation of selected slices could be omitted. Instead, both post‐marathon and follow‐up segmentations were obtained by automatic propagation of the baseline segmentation. This so‐called longitudinal propagation step, implemented with the ANTs library,^21^ as described by Ogier et al,^22^ consisted of a robust registration process with rigid and affine optimized transformations, followed by a diffeomorphic multi‐level registration with B‐spline regularization *ρ.* The registration parameters were: multi‐resolution levels = 4, gradient step = 0.1, shrink‐factors = 6 × 4 × 2 × 1, smoothing sigmas = 3 × 2 × 1 × 0 vox, number of iterations per level = 100 × 70 × 50 × 10, convergence threshold = 10^−6^, window size = 10 iterations. The knot spacing for the B‐spline smoothing was set at 26 mm at the base resolution level of the update displacement field and it was reduced by a factor of two for each succeeding multi‐resolution level. The described regularization *ρ* was applied at all dataset time points in order to correct for muscle shape deformations between two acquisition sessions, typically caused by difference in positioning and eddy currents. After the registration was completed, nearest‐neighbor interpolation was applied to restore the original integer values of the segmentation labels. All the computations were performed by means of an Intel Xeon E5‐2620 v4 processor.

### 2.6 Validation

To rate the quality of the supervised segmentation for the extraction of DTI indices, we considered several metrics. The volume similarity was assessed for each muscle in each dataset via the DSC^31^ for the propagated regions only, thus excluding the manually segmented slices used for input. To assess differences between manual and supervised automatic segmentations on the quantification of DTI indices, we focused on MD, FA and *λ*_3_. These DTI indices were calculated for all upper leg muscles using (i) the manual segmentations, (ii) the supervised automatic segmentations excluding the manually delineated slices and (iii) the full supervised automatic segmentations. The latter two were used to evaluate both the impact of the automatic propagation on DTI parameter estimations, as well as to consider the final complete supervised segmentation in comparison to the manual gold‐standard segmentation. Differences between manual and automatic segmentations were assessed by Bland‐Altman analysis and linear regression of the DTI indices.

### 2.7 Segmentation time

Individual muscles differ in their anatomical shape, aponeurosis locations, amounts of connective tissue and intra‐ and inter‐muscular fat. The muscle delineation complexity therefore varied between different muscles and between different subjects. In the transversal propagation, the operator performed the manual segmentations in a selected number of slices, as described above. Consequently, the time required for segmentation differed between muscles and was calculated from the number of slices that were manually delineated compared with the total number of slices in a given muscle. The mean values and the standard error of the time saved as a percentage are reported for each muscle.

## 3 RESULTS

**FIGURE 2:**
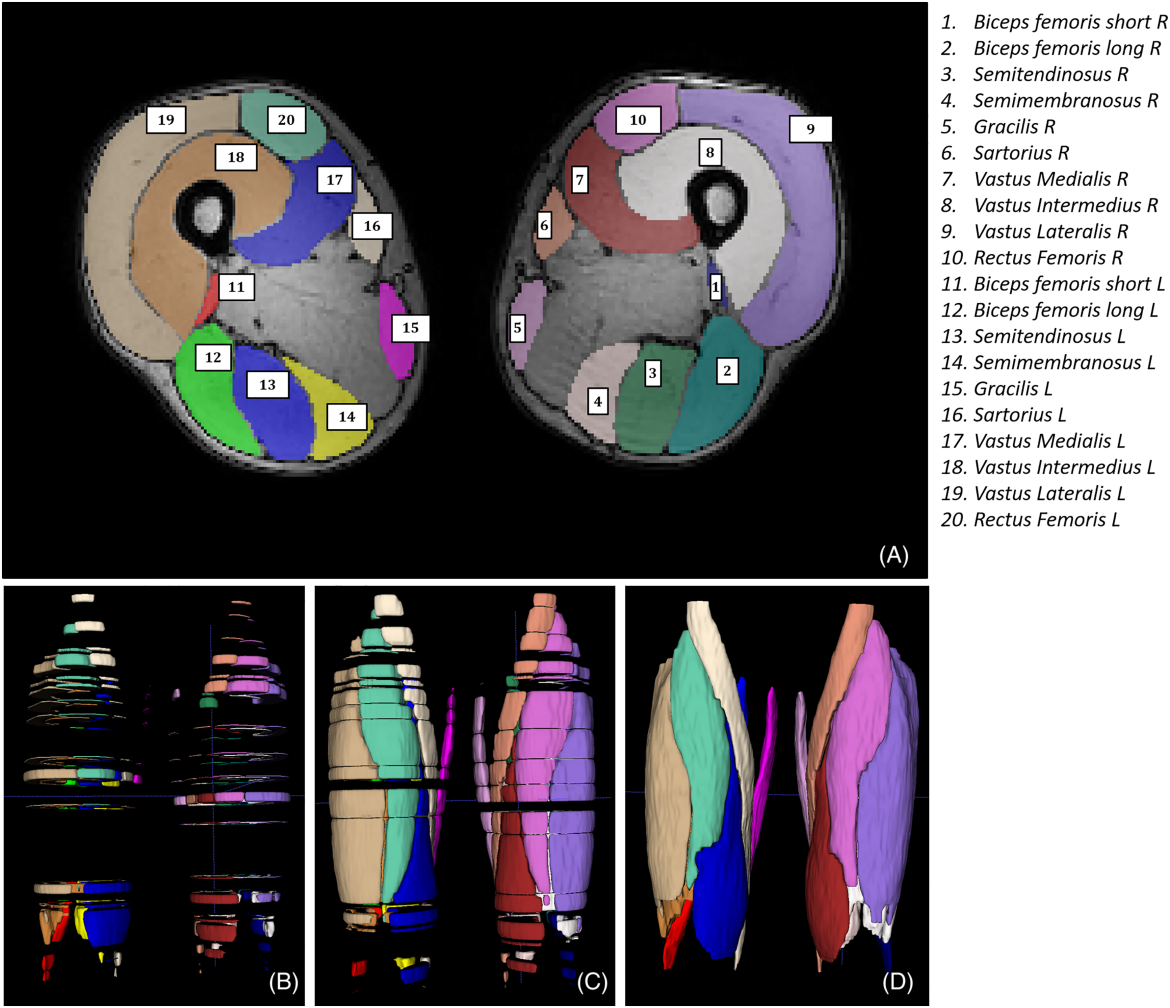
Anterior view of upper leg muscle segmentations, where knees are at the bottom and hips at the top of the image. (A) Transversal slice, approximately halfway between knee and hip, showing segmentations of the 10 muscles considered in this study in both legs, for example, 21 slices for the BFLH muscle in this subject. (B) Longitudinal views of the upper legs with an example of input segmentations at baseline. Adjacent slices were selected as input when muscle shape changed. (C) Propagations resulting from the transversal propagation step. (D) The full volumes obtained with the transversal propagation step

**FIGURE 3:**
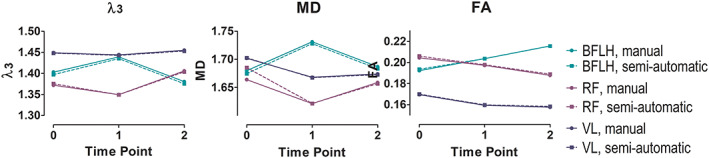
Comparison of the manual and semi‐automatic segmentations of the mean values of *λ*_3_, MD and FA for the BFLH, RF and VL muscles of a representative subject at baseline, post‐marathon and follow‐up time points

The transversal and longitudinal supervised segmentations were successfully executed for all 11 subjects. A transversal and longitudinal propagation of the segmentations of a representative subject are visualized in Figure 2, also indicating the manually segmented slices used as input for the transversal propagation. Figure 3 shows the comparison of the changes of the DTI indices between manual and supervised segmentations of three muscles for a representative subject at baseline, post‐marathon and follow‐up time points. Visual assessment of the graphs showed excellent correspondence for the DTI indices between manual and supervised automatic segmentations.

### 3.1 DSC values

**FIGURE 4:**
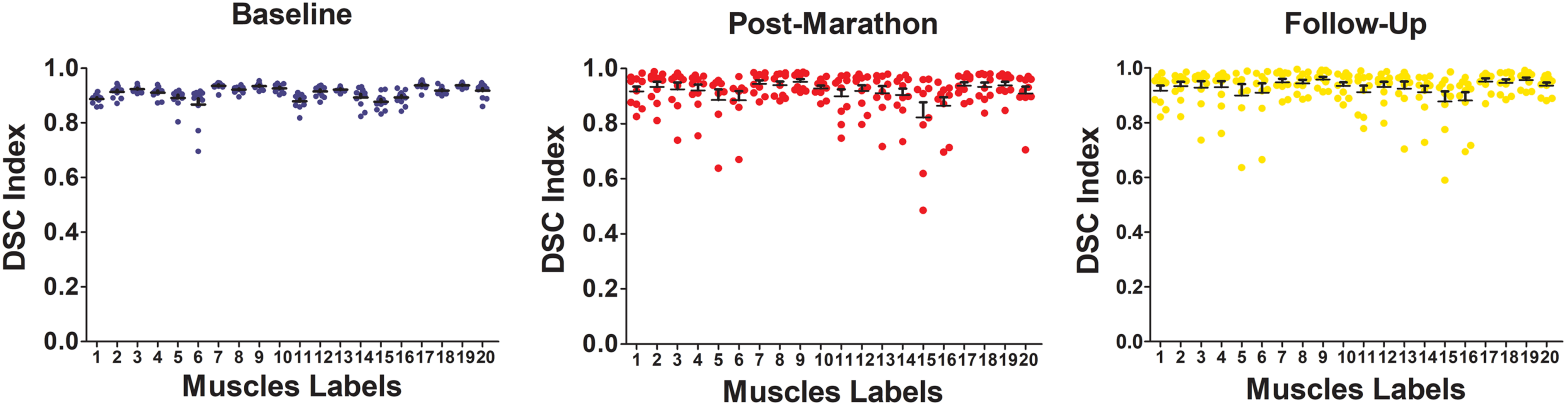
DSCs for the supervised segmentations compared with the manual segmentations for the baseline (4A in blue), post‐marathon (4B in red) and follow‐up (4C in yellow) *time points for all the segmented muscles. The numbers correspond to the muscles listed in Figure 2. Each dot reflects an individual subject and the group mean and standard deviation are shown in black*

DSC values calculated for the comparison between manual segmentation and the supervised segmentations of all analyzed muscles, excluding the manually segmented slices, for the three time points are shown in Figure 4. For the transversal propagation step, the DSC values for different subjects and muscles ranged between 0.62 and 0.95 (Figure 4 left), with 70% of the DSC values above 0.90. The DSC values for the volumes obtained through the longitudinal registration process ranged between 0.48 and 0.98 (with 68% above 0.90) for the post‐marathon time point and between 0.59 and 0.99 (with 76% above 0.90) at follow‐up (Figure 4 middle, right). In the transversal propagation the muscles that scored the highest and lowest DSC values were VI (0.94 ± 0.01) and G (0.87 ± 0.07) respectively. Similarly, in the longitudinal propagations the muscles with the highest and lowest DSC values were VI (post‐marathon 0.95 ± 0.03, follow‐up 0.96 ± 0.03) and G (post‐marathon 0.82 ± 0.16, follow‐up 0.88 ± 0.11).

### 3.2 Linear regression analysis

The G and S muscles were excluded from this part of the analysis because of low SNR (<15) in the diffusion images. The linear regression of mean *λ*_3_, MD and FA in the 16 muscle volumes (two legs, eight muscles each) obtained with supervised automatic segmentation versus the values obtained with manual segmentation, excluding the S and G muscles, resulted in *r* ^2^ values of 0.99 and *p* < 0.0001 for all indices at all time points (Figure 6 top, baseline; Figure 7 top, post‐marathon; Figure 8 top, follow‐up). By including the muscles with low SNR (S and G muscles) *r* ^2^ values were lower, 0.98 (*λ*_3_), 0.91 (MD) and 0.99 (FA) at baseline, respectively (*p* < 0.0001), whereas at post‐marathon *r* ^2^ values were 0.96 (*λ*_3_), 0.91 (MD) and 0.94 (p < 0.0001) and at follow‐up *r* ^2^ values were 0.95 (*λ*_3_), 0.95 (MD) and 0.99 (FA) (*p* < 0.0001).

### 3.3 Bland‐Altman analysis

Bland‐Altman analysis were carried out for the three different time points separately by considering the mean values of MD, FA and *λ*_3_ of each segmented muscle with sufficient SNR of each participant. The derived 95% limits of agreement (LoAs) are reported in <TAB 1>Table 1. At baseline, the 95% LoA ranged between 0.5% and 1.5% in the full volumes and between 0.7% and 3% excluding manual segmentations (Figure 6 bottom). The bias ranged between −0.0008 and 0.0010. At post‐marathon and follow‐up, the LoA ranged between 0.7% and 3.1% (Figure 7 bottom and Figure 8 bottom) with the bias between −0.0005 and 0.0016. The best agreement was presented by *λ*_3_. At baseline, some distinct outliers were observed in the Bland‐Altman plots (Figure 7). Outliers in *λ*_3_ corresponded to the BFLH right leg. Some outliers in MD were generated by the right and left SM and the left RF. When including the manually segmented slices no outliers were observed.

**TABLE 1:** 95% LoAs, the LoA percentage with respect to the mean resulting from the Bland‐Altman analysis and the bias of the DTI parameters of the supervised and manual muscle segmentations at the THREE time points

DTI parameter	Baseline full volume			Baseline excluding manual segmentations			Post‐marathon			Follow‐up		
*λ_3_ (10^−3^ mm^2^/s)*	[−0.008 0.008]	0.5%	0.0003	[−0.016 0.020]	0.7%	0.0010	[−0.016 0.019]	0.7%	0.0016	[−0.012 0.014]	0.7%	0.0011
MD (10^−3^ mm^2^/s)	[−0.023 0.023]	1.2%	−0.0008	[−0.030 0.031]	1.8%	0.0002	[−0.026 0.031]	1.7%	0.0023	[−0.032 0.034]	1.8%	−0.0003
FA (—)	[−0.003 0.003]	1.5%	−0.0003	[−0.006 0.005]	3.0%	−0.0004	[−0.006 0.005]	3.0%	−0.0005	[−0.006 0.005]	3.1%	0.0007

### 3.4 Input slices and segmentation time

**FIGURE 5:**
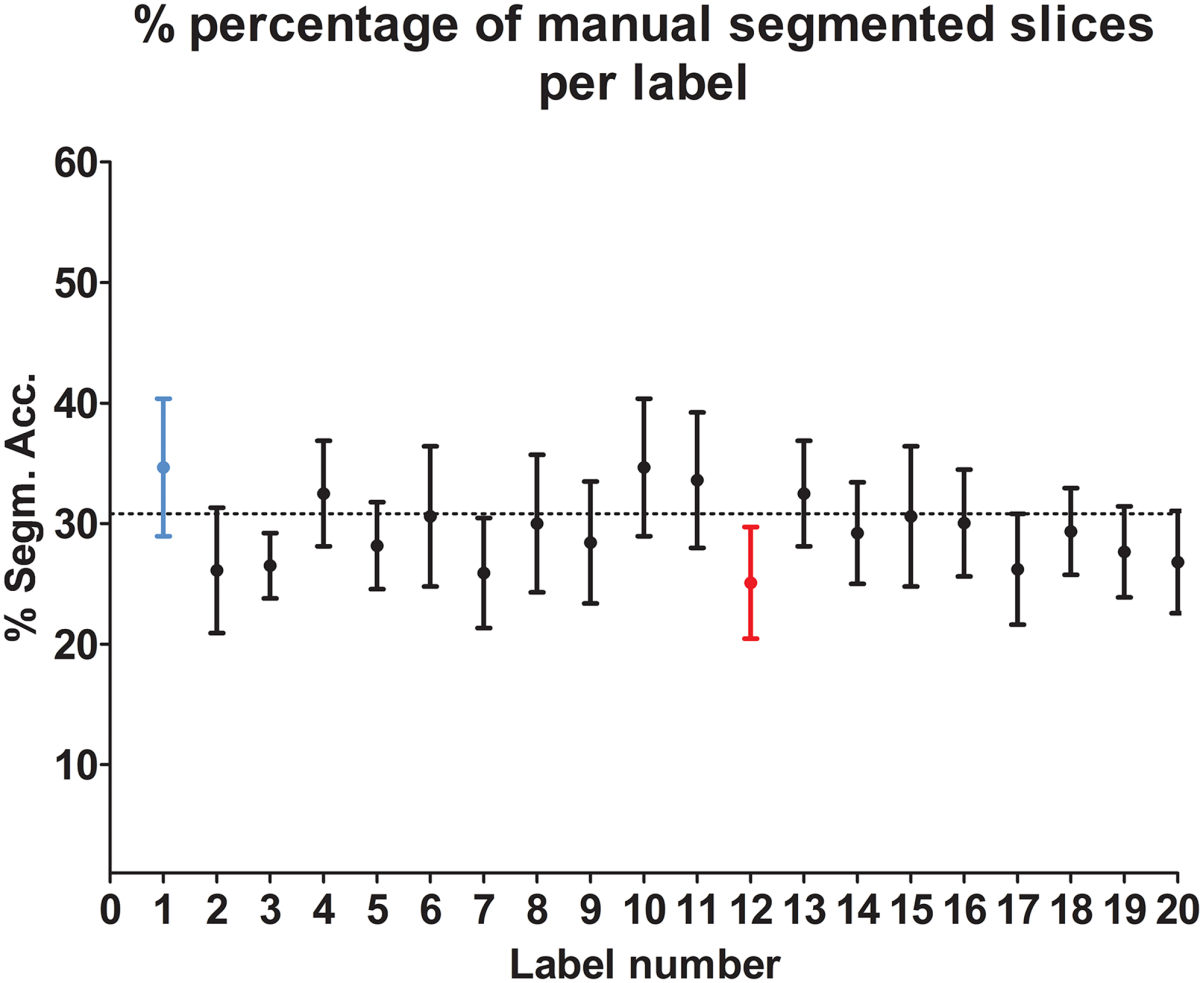
Percentage of segmented slices used for transversal propagation at baseline for the individual muscles. The BFSH of the right leg (blue) required the most manual segmented slices; the BFLH of the left leg (red) the fewest. Each point represents the mean and the range of the segmentation acceleration. The dotted line represents the average acceleration time

The percentage of slices for which manual segmentation was required at baseline, prior to transversal and longitudinal propagation, is shown in Figure 5. On average only 30% of the slices needed to be manually segmented at baseline, thus resulting in more than three times faster segmentations at baseline and 10 times faster for the three time points. The BFSH of the right leg required the most manual segmented slices, the BFLH of the left leg the fewest. Figure 5 shows that the numbers of manually segmented slices of the same muscle in the right and left legs are in the same range. Furthermore, for the longitudinal segmentation the reduction in time is even greater, as these required no additional manual segmentations.

**FIGURE 6:**
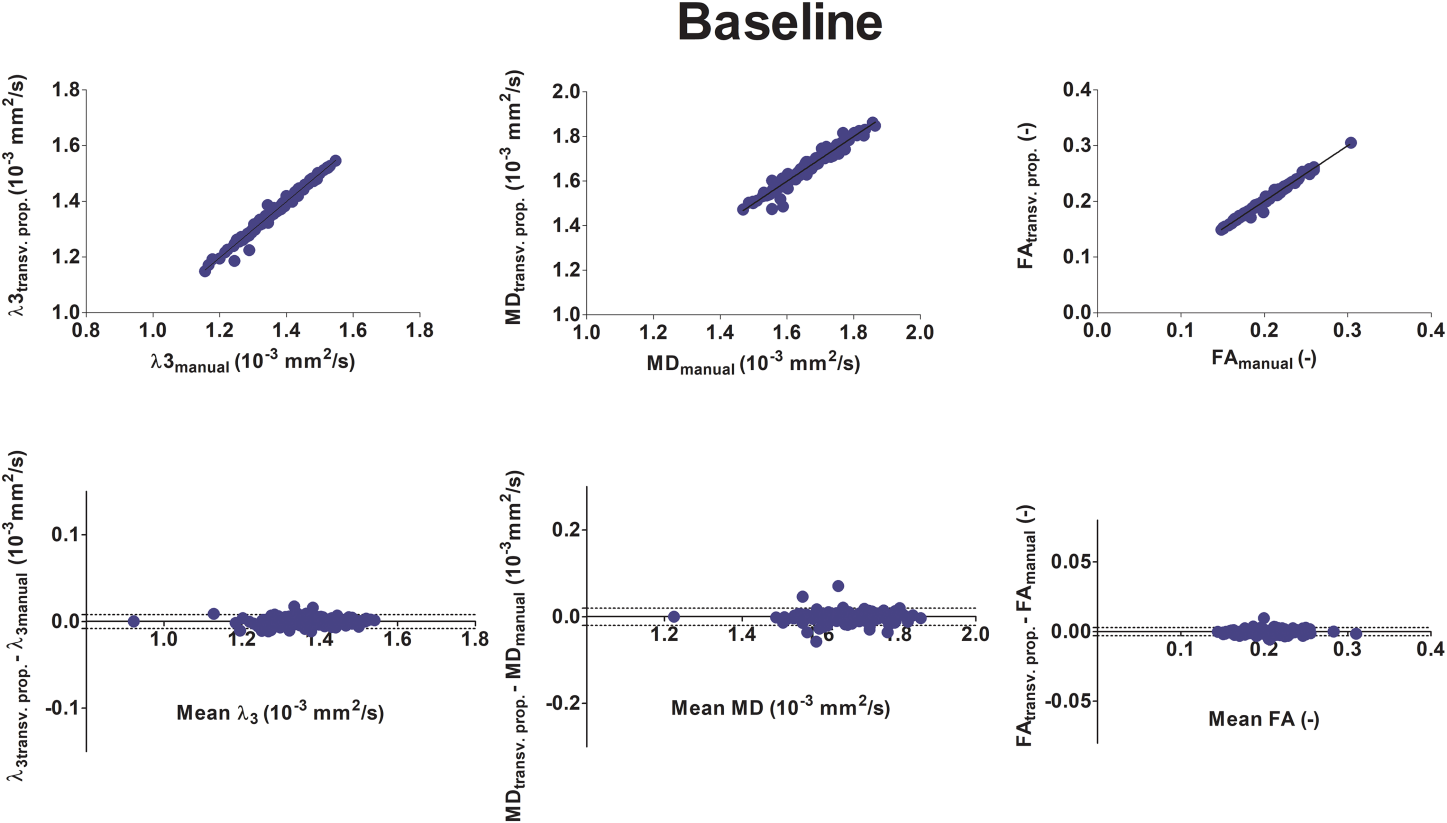
Linear regression analysis (top) and Bland‐Altman plot (bottom) at baseline of diffusion indices λ_3_ (10^−3^ mm^2^/s), MD (10^−3^ mm^2^/s) and FA (—) comparing manual segmentation with those resulting from the transversal propagations

**FIGURE 7:**
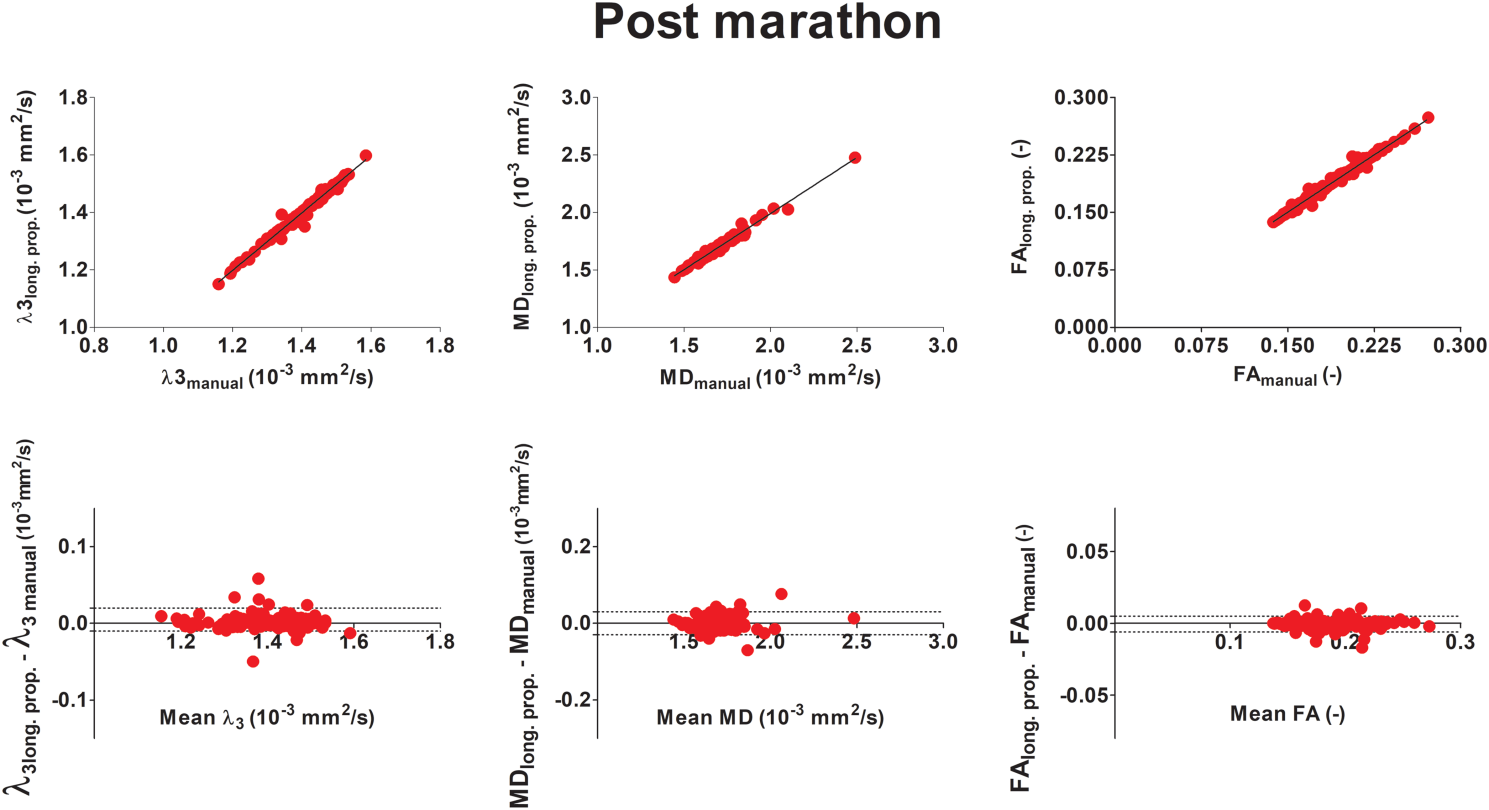
Linear regression analysis (top) and Bland‐Altman plot (bottom) post‐marathon of diffusion indices λ_3_ (10^−3^ mm^2^/s), MD (10^−3^ mm^2^/s) and FA (—) comparing manual segmentation with those resulting from the longitudinal propagations

**FIGURE 8:**
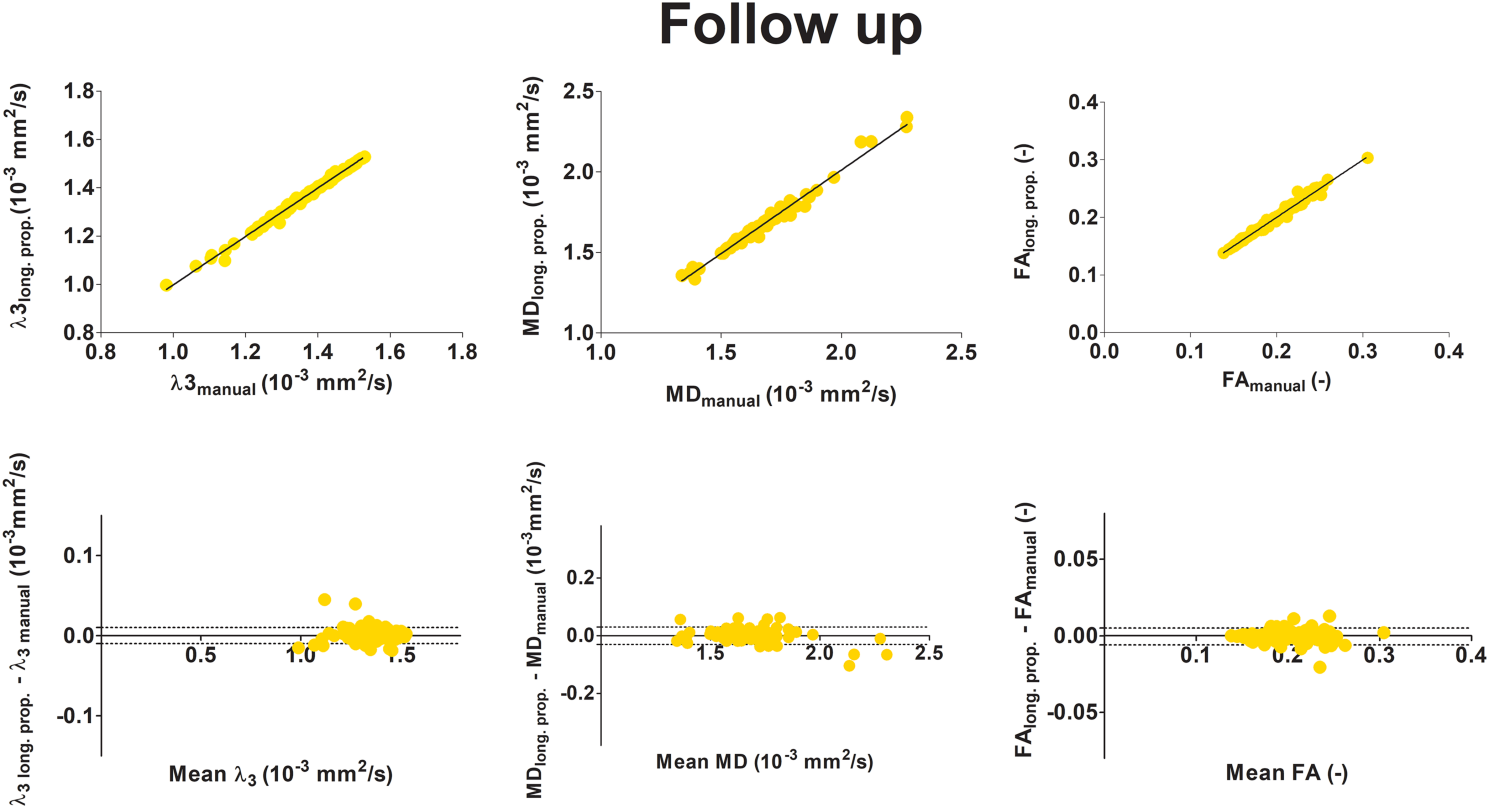
Linear regression analysis (top) and Bland‐Altman plot (bottom) at follow‐up of diffusion indices λ_3_ (10^−3^ mm^2^/s), MD (10^−3^ mm^2^/s) and FA (—) comparing manual segmentation with those resulting from two longitudinal propagation steps

## 4 DISCUSSION

DTI is becoming increasingly popular to study muscle injury and disease, as the diffusion indices provide a direct window into muscle fiber integrity and architectural organization.^4^, ^5^ For quantitative analysis of changes in diffusion values, segmentation of individual muscles is required. Such segmentations are often performed manually,^12^ which is a time‐consuming, laborious and an operator‐dependent process. This study aimed to evaluate the impact of a supervised semi‐automatic segmentation framework on the quantification of DTI indices in the upper leg muscles. Compared with the conventional manual segmentation approach, similar DTI indices were found using this supervised semi‐automatic segmentation framework, as represented by high LoAs and *r* ^2^ values. Furthermore, this study showed that the segmentation time of the entire muscle volume can be three times faster for a single time point without significant impact on the quantification of DTI indices, which we believe is an important step to more widespread application of DTI to study muscle injury and disease. In addition, the time saving for multiple time points is even larger (10‐fold for three time points), given that no further manual delineations are needed.

The Dice similarity coefficient was, with a few outliers, consistently high when comparing manual and supervised segmentations. These results agree with the previous results that were obtained by Ogier et al,^22^ who reported a DSC of 0.91 for the transversal propagation and 0.88 for the longitudinal propagation. Interestingly, for most of our muscles, with the exception of the smaller muscles with a more changeable cross‐sectional area in the proximal‐distal direction (S and G muscles), even better similarity indexes were found. We have not yet characterized with certainty the reasons why some muscles are less well propagated at post‐marathon and follow‐up in comparison with baseline, but the factors that might have influenced the results are the differences in the semi‐automatic and automatic segmentation methods applied, the muscle shape deformation between two acquisition sessions and the muscle size, because both G and S, the two smallest muscles in the upper leg, showed the lowest Dice coefficient. However, more importantly, in this longitudinal study we also evaluated the impact of automatic segmentation on the quantification of the muscle DTI indices. The Bland‐Altman analysis resulted in excellent LoA values (maximum of 3% for FA) and low bias (maximum of 0.4% for FA in the transversally propagated volumes). These ranges can be compared with the changes in muscle DTI parameters that are normally expected in muscle injury and disease. For example, changes in DTI indices reported after physical activity, such as a triathlon^8^ or a marathon,^9^ are generally larger than the 95% LoAs reported above. Also, the changes in DTI indices observed in the presence of musculoskeletal diseases, as shown in the study by Maggi et al^7^ in patients with muscular dystrophy (FA varies by 8.4%), are larger than the 95% LoA found in our work. In the study by Sigmund et al^6^ in dermatomyositis patients, MD in the quadriceps muscles varied by 1.7%, which is smaller than the 95% LoA we found over all analyzed muscles. However, in our work the quadriceps muscles showed the best correspondence with manual segmentation. Consequently, when considering our LoA for quadriceps muscles only, even these changes could be quantified. However, this would not be the case for the other muscle groups. This indicates that by using the proposed semi‐automatic segmentation method the changes in DTI indices that are due to physical activity and certain diseases, such as muscular dystrophy, remain detectable, whereas smaller changes due to other pathologies, such as dermatomyositis, cannot be detected in all muscle groups.

It is furthermore interesting to review some of the input requirements and time savings using this supervised semi‐automatic segmentation framework. At baseline, only 30% of the slices were required as input for the supervised segmentation compared with the full manual segmentation, reducing the time spent for manual delineation of muscle outlines for a full segmentation of 10 muscles in both upper legs from 840 min to 140 min. Furthermore, the segmentations of the post‐marathon and follow‐up time points were obtained directly from the baseline, without additional need of manual delineation of the muscles. This makes the segmentation process in larger cohort studies much more manageable, especially in longitudinal studies. However, for this comparison, we did not include the computer processing time for the transversal and longitudinal propagation steps that can be executed without further user interaction in the background. These steps do require time, which strongly depends on the computer processor (ie of the order of 120 min for the transversal propagation and 300 min for the longitudinal propagation using an Intel Xeon E5‐2620 v4 processor). For the transversal propagation at baseline it was important that a sufficient number of slices were manually segmented in those regions with large changes in muscle cross‐section, typically at the muscle origin and insertion. The selection of these slices based on muscle shape variations is a subjective factor in the transversal segmentation process that could lead to user‐dependent performance. In fact, in comparison with the study by Ogier et al,^22^ for the sake of robustness we segmented a higher percentage of slices manually than strictly necessary, leading to less reduction in segmentation time (70% in our study as compared with 85% by Ogier et al). This difference is mainly due to the fact that in this study the semi‐automatic segmentation framework was applied to segment the full muscle volume, while in the study by Ogier et al the most distal muscle regions (10 cm above the knees) were not segmented.

Over the past years, some other semi‐automatic and fully automated segmentation methods for skeletal muscle have been proposed. These methods use non‐rigid multi‐atlas registration or convolutional neural networks (CNNs) and have primarily focused on specific locations in the upper leg muscles^13^, ^14^, ^15^ rather than full volumetric analysis.^16^ Furthermore, these methods have also been used to determine muscle volume and to extract fat fractions. One of these previous studies, by Kemnitz et al,^14^ compared active shape model (ASM) and active contour model (ACM) approaches at mid‐thigh level and showed that the precision of the technique was higher for the quadriceps muscles (DSC values of 0.93 and 0.94) than for the hamstring muscles (DSC values of 0.87 and 0.89) and lowest for the S muscle (DSC values of 0.74 and 0.82). These findings are in agreement with our results, where we found high DSC values for the quadriceps and hamstring muscles and lowest for the G and S muscles. The CNN approach proposed by Kemnitz et al^13^, ^15^ proved to be the fastest approach, less than 1 s per slice, and was also very precise (average DSC value 0.98). However, it is important to note that both methods described above have been trained only for a particular anatomical location rather than for the segmentation of the full volume of individual muscles; moreover, no volumetric analysis was reported. Another approach that has been used is the AMRA automatic segmentation technique developed at Linkoping University. This approach is based on non‐rigid multi‐atlas registration on water fat Dixon images^18^ and was used to evaluate automatic quantification of fat fraction and volume increases in the quadriceps muscle group.^16^ This study^18^ showed very good correlation with the manual segmentation of the estimated volumes of the quadriceps muscles, with *r* = 0.98 and *p* < 0.0001. These values are in the same range as what we found for the DTI indices determined in individual muscles rather than in a muscle group. Unfortunately, no information is given concerning other muscle groups or individual muscles.

This study has some limitations. First, the manual segmentation of the input slices was performed only once, by two albeit expert observers, because the manual segmentation is very time consuming and laborious, and requires quite some experience and anatomical knowledge. Therefore, the intra‐observer reproducibility was not tested at this point. Second, thus far, we have only focused on the impact of supervised segmentation on DTI quantification using whole muscle segmentations. The impact of semi‐automatic segmentation on quantification could vary along the proximo‐distal muscle axis and therefore affect more localized assessments in a different manner. Additionally, in this work only healthy muscle tissue has been evaluated. Pathophysiological changes due to injury or disease alter image contrast and could impact the performance of the supervised semi‐automatic segmentation framework. Future studies will include localized muscle damage to evaluate how well the propagation algorithms perform under these circumstances.

## 5 CONCLUSIONS

The purpose of the present study was to evaluate a supervised muscle segmentation framework developed by Ogier et al,^21^, ^22^ for the quantification of DTI indices in individual muscles of the thighs. Using this tool, we assessed the accuracy, feasibility and impact on the quantification of DTI indices in comparison with manual segmentation as well as the reduction in work load compared with manual segmentation. Linear regression and the Bland‐Altman analysis of the DTI indices showed good agreement between the results obtained with manual segmentation and the results obtained with the supervised muscle segmentation framework. The work load and segmentation time were reduced threefold at baseline compared with manual segmentation and the segmentation of post‐marathon and follow‐up time points was completely automated. The proposed semi‐automatic segmentation method for the detection of changes in DTI indices that are due to physical activity and diseases proved fast, feasible, accurate, reproducible and less operator dependent.

## FUNDING INFORMATION

Sportinnovator grant of The Netherlands Organization for Health Research and Development, ZonMw (50‐53800‐98‐PR020).

Dutch Technology Foundation TTW (DIMASK 15500).
